# Factors Associated With Engagement With a Web-Based Lifestyle Intervention Following Provision of Coronary Heart Disease Risk: Mixed Methods Study

**DOI:** 10.2196/jmir.7697

**Published:** 2017-10-16

**Authors:** Juliet A Usher-Smith, Laura R Winther, Guy S Shefer, Barbora Silarova, Rupert A Payne, Simon J Griffin

**Affiliations:** ^1^ The Primary Care Unit Department of Public Health and Primary Care University of Cambridge Cambridge United Kingdom; ^2^ Global Health Section Department of Public Health University of Copenhagen Copenhagen Denmark; ^3^ Faculty of Health, Social Care and Education Anglia Ruskin University Cambridge United Kingdom; ^4^ MRC Epidemiology Unit Institute of Metabolic Science University of Cambridge Cambridge United Kingdom; ^5^ Centre for Academic Primary Care University of Bristol Bristol United Kingdom

**Keywords:** Web-based intervention, cardiovascular disease, engagement, risk, qualitative research

## Abstract

**Background:**

Web-based interventions provide the opportunity to combine the tailored approach of face-to-face interventions with the scalability and cost-effectiveness of public health interventions. This potential is often limited by low engagement. A number of studies have described the characteristics of individuals who engage more in Web-based interventions but few have explored the reasons for these variations.

**Objective:**

We aimed to explore individual-level factors associated with different degrees of engagement with a Web-based behavior change intervention following provision of coronary heart disease (CHD) risk information, and the barriers and facilitators to engagement.

**Methods:**

This study involved the secondary analysis of data from the Information and Risk Modification Trial, a randomized controlled trial of a Web-based lifestyle intervention alone, or alongside information on estimated CHD risk. The intervention consisted of three interactive sessions, each lasting up to 60 minutes, delivered at monthly intervals. Participants were characterized as high engagers if they completed all three sessions. Thematic analysis of qualitative data from interviews with 37 participants was combined with quantitative data on usage of the Web-based intervention using a mixed-methods matrix, and data on the views of the intervention itself were analyzed across all participants.

**Results:**

Thirteen participants were characterized as low engagers and 24 as high engagers. There was no difference in age (*P*=.75), gender (*P*=.95), or level of risk (*P*=.65) between the groups. Low engagement was more often associated with: (1) reporting a negative emotional reaction in response to the risk score (*P*=.029), (2) perceiving that the intervention did not provide any new lifestyle information (*P*=.011), and (3) being less likely to have reported feeling an obligation to complete the intervention as part of the study (*P*=.019). The mixed-methods matrix suggested that there was also an association between low engagement and less success with previous behavior change attempts, but the statistical evidence for this association was weak (*P*=.16). No associations were seen between engagement and barriers or facilitators to health behavior change, or comments about the design of the intervention itself. The most commonly cited barriers related to issues with access to the intervention itself: either difficulties remembering the link to the site or passwords, a perceived lack of flexibility within the website, or lack of time. Facilitators included the nonjudgmental presentation of lifestyle information, the use of simple language, and the personalized nature of the intervention.

**Conclusions:**

This study shows that the level of engagement with a Web-based intervention following provision of CHD risk information is not influenced by the level of risk but by the individual’s response to the risk information, their past experiences of behavior change, the extent to which they consider the lifestyle information helpful, and whether they felt obliged to complete the intervention as part of a research study. A number of facilitators and barriers to Web-based interventions were also identified, which should inform future interventions.

## Introduction

Noncommunicable diseases have now overtaken communicable diseases in causing the greatest disease burden worldwide, with coronary heart disease (CHD) being the number one cause of disability-adjusted life years globally [1]. Four modifiable lifestyle risk factors (tobacco use, high alcohol consumption, unhealthy diet, and low levels of physical activity) have been associated with 80% of deaths caused by cardiovascular disease (CVD) [2]. This finding has led to an increasing focus on affordable effective behavior change interventions, including collective approaches that aim to shift the entire population distribution of risk factors, and approaches that focus on individuals.

With the expanse and scope of the Internet, Web-based interventions provide the opportunity to combine the tailored approach of face-to-face interventions with the scalability and cost-effectiveness of public health interventions, and are potentially appealing to the public because they are convenient and easily accessible [3,4]. Systematic reviews have shown that Web-based interventions have the potential to influence behavior [5-7]. However, this potential is often limited by low levels of engagement and high rates of attrition [8,9]. Understanding why some individuals engage in Web-based interventions whilst others do not is important to optimize future interventions.

A number of quantitative studies have described the characteristics of individuals who engage more in Web-based behavior change interventions. The findings have been mixed, with one study finding no association between website use and clinical and sociodemographic variables [10], whilst others have reported higher engagement in younger people and those with higher mental health scores, higher perceptions of general health, higher perceived risk, lower income, and in less than full-time employment [11,12]. To our knowledge, only one qualitative study has explored the reasons for these differing levels of engagement and that study only reported reasons given by women who had not logged onto a Web-based intervention providing information about risks of breast cancer [11]. More research is needed to better understand the factors (at an individual level) that are associated with engagement with Web-based health behavior interventions.

A number of behavior change theories additionally suggest that lifestyle interventions will only be successful if individuals perceive themselves to be at risk of developing the target disease [13,14]. This factor has led to the incorporation of risk communication into many major clinical guidelines for routine practice [15-19] and the English National Health Service (NHS) Health Checks program, which aims to assess CVD risk for individuals aged 40-74 years without preexisting CVD [20]. Whilst the evidence for any impact of risk communication on behavior change is limited [21], very little is known about the impact of risk communication on subsequent engagement with Web-based health behavior interventions.

The Information and Risk Modification (INFORM) Trial [22] was a randomized controlled trial comparing the impact of providing phenotypic and genetic CHD risk scores alongside a Web-based lifestyle intervention. In additional to behavioral outcomes, INFORM included quantitative measurements of engagement with the Web-based lifestyle intervention and a nested qualitative study with face-to-face individual interviews with participants throughout the trial [23]. The aims of this study were to use the data from the face-to-face interviews to explore the factors associated with different levels of engagement with the Web-based intervention, and the barriers and facilitators to engagement in general.

## Methods

### Participants and Setting

This study is a secondary analysis of data collected as part of the INFORM trial. Details of that trial are reported elsewhere [22]. In brief, INFORM was a parallel group randomized controlled trial that aimed to explore the short-term effects on health-related behaviors of giving people different types of information online about their estimated risk of CHD in the subsequent 10 years, together with Web-based lifestyle advice. A convenience sample of 956 blood donors aged 40-84 years from across England who took part in the INTERVAL study [24] with no previous history of CVD were allocated to either no intervention (control group), or to one of three active intervention groups: Web-based lifestyle advice only; Web-based lifestyle advice plus information on estimated 10-year CHD risk as a percentage, heart age (the chronological age of someone with the same absolute risk of CHD but with healthy risk factors), and comparison with someone of the same age and gender who had a healthy lifestyle based on phenotypic characteristics; and Web-based lifestyle advice plus information on estimated 10-year CHD risk, heart age, and healthy comparison based on phenotypic and genetic characteristics.

### The Web-based Intervention

The Web-based lifestyle advice was based on an intervention originally developed for the Heart to Health study, which was shown to be effective in a randomized controlled trial [25]. The advice consisted of a library of over 250 webpages providing advice on physical activity, diet, smoking, and medication tailored to the participants’ responses to a prestudy questionnaire and choice of risk-reducing strategies. The intervention was delivered through three interactive sessions at monthly intervals, each lasting up to 60 minutes. Prior to the first session, participants were presented with their 10-year CHD risk information and asked to choose to take part in any or all of the modules related to diet, physical activity, and smoking cessation. The first session then began with education on either diet, physical activity, or smoking cessation alongside tips on how to overcome self-identified barriers to risk reduction and the creation of steps toward self-identified actionable goals. The second and third sessions included similar content, with participants beginning by reviewing their progress toward goals, continuing with education, and tips to overcome barriers, and then finishing with identification of new goals [22].

### Qualitative Data

Face-to-face interviews with a purposive sample of 41 participants were conducted as part of the INFORM trial by an experienced qualitative researcher (GS). Full details of the recruitment and methods are reported in detail elsewhere [23]. Briefly, in order to sample participants who could provide the richest data on the primary trial question, participants who received medium to high risk scores (a 10-year CHD risk >10% or heart age at least two years older than their real age) were mainly selected. In this study, we only included the 37 participants who had received either a phenotypic or phenotypic plus genotypic risk score. Each interview lasted between 30-45 minutes and was guided by a schedule covering the participants’ understanding of CHD risk, their reaction to receiving a risk score, their intentions to change behavior, their attempts at actually changing behavior, and their experience of the Web-based intervention. All interviews were audio-recorded and professionally transcribed.

### Quantitative Data

Quantitative data on usage of the Web-based intervention was collected by tracking which pages participants had accessed during the trial. Participants were considered high engagers with the website if they completed all three sessions for either diet, physical activity, or smoking, and low engagers if they did not. Student’s t-tests or Chi-squared tests were used to assess differences between the high and low engagers with significance set at *P*<.05.

### Analysis

We first used thematic analysis [26] to analyze the qualitative data from the interviews undertaken within the INFORM study. Using an inductive approach, after repeated reading of the transcripts, three members of the team (LW, GS, and JUS) developed a coding framework from the empirical data focusing on how people reacted to and assigned meaning to risk information, their prior experiences of health behavior change, their engagement with the Web-based intervention, and their views on the intervention itself. This framework was independently piloted on four transcripts by two researchers (LW and GS) to ensure a consistent approach to coding. The coding of the remaining transcripts was then completed by one researcher (LW) using NVivo software.

Once coding was complete, we combined the qualitative data with the quantitative data in a mixed-methods matrix with one row for each of the 37 participants. Data on the level of website engagement was used to divide participants based on whether they were low or high engagers and Chi-square tests were used to test associations. After identifying themes associated with engagement with the website from this matrix, we then returned to the qualitative data to explore those themes in greater depth. Data on the views of the intervention itself were also analyzed separately across all participants using thematic analysis.

## Results

### Participant Characteristics

The characteristics of the 37 participants are described in Table 1. The majority of individuals were married with university degrees and in the high-income category. The mean phenotypic risk was 12.6% for men (range 4-62%) and 3.8% (range 0.6-11%) for women and the mean genotypic risk was 12.6% (range 5-28%) for men and 4.1% (range 0.5-15%) for women.

### Qualitative Themes Associated With Low Engagement

Using the quantitative data from the website, 13 participants were characterized as low engagers and 24 as high engagers. There was no difference in age (*P*=.75), gender (*P*=.95), or the difference between the estimated phenotypic heart age the participants received and their chronological age (*P*=.65) between the groups. Table 2 shows a section of the mixed-methods matrix ordered according to the level of website engagement.

Low engagement with the website was more often associated with: (1) reporting a negative emotional reaction to the risk score (*P*=.029), (2) perceiving that the intervention did not provide any new lifestyle information (*P*=.011), and (3) being less likely to have reported feeling an obligation to complete the intervention as part of the study (*P*=.019). The matrix also suggested an association between low engagement and less success with previous behavior change attempts, although the statistical evidence for this was weak (*P*=.16). No associations were seen between engagement with the website and barriers or facilitators to health behavior change, or comments about the design of the intervention itself. In the latter case, participants in both groups described aspects of the intervention which they thought were helpful or unhelpful, but whether they chose to engage with the intervention or not appeared to be dominated by other factors.

**Table 1:** Characteristics of participants.

Participant characteristic		n=37
**Gender**		
	Male	23
	Female	14
**Age at baseline (years)**		
	40-49	5
	50-59	14
	60-69	13
	70-80	5
**Study group**		
	Phenotypic risk + genetic risk + lifestyle advice	22
	Phenotypic risk + lifestyle advice	15
**Marital status**		
	Married	26
	Separated or divorced	3
	Widowed	3
	Single	5
**Level of education**		
	No formal education	1
	Secondary education (to age 18)	17
	University education	19
**Annual income**		
	Less than £8000	1
	Between £8001-40,000	13
	More than £40,000	19
	Did not know or did not answer	4
**Estimated phenotypic 10-year CHD risk**		
	<5%	11
	5-10%	14
	10-20%	9
	>20%	3

**Table 2:** Mixed-methods matrix ordered according to the level of website engagement, where dots indicate the presence of that theme within the qualitative interview data.

Level of engagement	Participant characteristics			Response to risk information	Previous behavior change attempts		Views of the intervention	
	ID	Age	Sex	Negative emotional reaction	Unsuccessful	Successful		No new information	Felt obliged to complete
Low engagers	9	73	F					●	
	12	69	M	●	●			●	
	13	64	M					●	
	15	56	M	●	●			●	
	19	75	F					●	
	22	59	M	●				●	
	24	55	M	●	●			●	
	25	67	M			●		●	
	26	44	F	●	●			●	
	27	54	F	●		●		●	
	30	56	M	●				●	
	31	44	F		●			●	
	33	59	M	●				●	
High engagers	1	64	M					●	
	2	70	M						
	3	57	M					●	
	4	59	F		●				
	5	72	M	●		●			●
	6	63	F					●	
	7	57	M	●		●		●	
	8	67	M			●		●	
	10	68	M			●		●	
	14	68	F						
	16	63	M		●				
	17	64	F					●	●
	18	61	M	●	●				
	20	49	M			●		●	●
	21	55	M	●					
	23	76	M			●		●	●
	28	58	M						
	29	64	M			●		●	
	32	66	M					●	●
	34	51	F	●		●		●	●
	35	55	F	●				●	●
	36	56	F		●	●		●	
	37	46	F		●				
	40	45	F		●			●	●

#### 1) A Negative Emotional Reaction to the Risk

A greater proportion of low engagers described a negative emotional reaction to the risk, which was understood as expressing fear, anxiety, worry, shock, concern, or irritation when being asked during the interview to recall their feelings at the time they received the risk information. In many cases this reaction was surprise, disappointment, or worry because the risk did not match how they perceived themselves in relation to their health behavior and comparison with others:

It was a bit of a shock to be honest, because as I say, I thought that when I would get the results of that my, say, I’m 59, I know, but I thought my heart would be, or my rating would be say down much lower at 54, 55 or something like that...’cos of the amount of exercise I do and, you know, my weight I think is about right and I’m, I don’t get ill at all and fortunately I haven’t got any, you know, any long-term health problems.

I22 – Male, aged 59, low engager

Yeah, my heart age was...It was about seventy I think and I’m fifty-five and that was, you know, it was worrying, especially considering I’ve never smoked or anything like that...I thought, well rather than having twenty-five years maybe, hopefully, I might only have ten or less.

I24 – Male, aged 55, low engager

In some cases, particularly amongst those participants who did not fully understand the risk information, this led to confusion, irritation, or annoyance.

Yeah, it was [confusing] actually, because it just came, it didn’t explain why it would be that way so I mean I did, I haven’t angst, I haven’t sort of lost sleep over it but I did kind of think why basically, why should it be that way? The percentages were pretty much the same which seemed bizarre given the differential on the age thing.

I27 – Female, aged 54, low engager

By comparison, several high engagers had also felt irritated, surprised, or concerned by receiving risk scores higher than they had expected but, unlike the low engagers, described acceptance of the score as a reasonable assessment.

Well, pretty irritated really, but since it was based on answers I’d given, and I’d given them fairly honestly, I mean I had no, you know, it, it’s an algorithm that you’ve applied to the information I gave so I could question the, I can’t question the information, I could question the algorithm but I wasn’t going to. I, I took it as being a reasonable assessment of probabilities or of causal factors.

I18 – Male, aged 61, high engager

Useful and concerned ’cos I think 60, a heart age of 69 is significantly greater than I would like it to be, so that’s why I read on all the material about diet and exercise because I wanted to see if I could do something about it.

I21 – Male, aged 55, high engager

#### 2) Reporting That the Intervention Did Not Provide Any Helpful Lifestyle Information

Notably all of the low engagers reported that the intervention did not provide any new lifestyle information.

*I don’t think I learnt anything new, it just told me what I could do and, to be fair, what I know I could do, you know, or know what I should do, I don’t think I learnt anything more.*

I15 – Male, aged 56, low engager

Whilst many of the high engagers also felt there was little new lifestyle information, some of those nevertheless considered that the intervention was still helpful as it presented the lifestyle information differently or reinforced their prior knowledge.

No [I did not learn anything new], I think I was aware of it, but it’s when, you know, you see it linked up, because you, so much information comes out about diet, food, and it does change quite regularly, sometimes it’s difficult, it is difficult to try and keep up with everything.

I20 – Male, aged 49, high engager

I think it reinforced what I was aware of, and I think it’s always good to keep refreshing, because things might change a little. So, I don’t think I thought that there was any “wow” in it, but it was, yeah, yeah, okay that’s fine.

I3 – Male, aged 57, high engager

#### 3) Not Feeling Obliged to Complete the Intervention

A further theme associated with level of engagement was the finding that many of the high engagers had completed all three sessions partly for the purpose of the study. For these participants, any reactions they had to the risk information or views about the intervention were superseded by a desire to “do what they had been told” or committed to.

I thought having been asked to do it you know, I’d religiously go through it and make sure you know, I’d covered all the elements.

I32 v Male, aged 66, high engager

*I’m the sort of person who does do, I mean as I say, I tend to do what I’m told, having signed to do this, I will do it and I will do every module.*

I35 – Female, aged 55, high engager

#### 4) Less Success With Previous Behavior Change Attempts

Although not statistically significant, the final theme found amongst low engagers related to prior experiences of behavior change. Compared to high engagers, low engagers tended to have had more unsuccessful prior behavior change attempts and less successful experiences.

...the, the eating habit I’ve got, that’s going to be my biggest problem, I bring a banana into work and then I, five o’clock, oh, it’s still there, and I’ve walked down to the shop and got myself a roll [laughs]. So, changing that is my bigger problem, the eating part, although I have been on a diet in the past and lost nearly four stone, but then it all came back again.

I15 – Male, aged 56, low engager

Well when I, when I discovered my, I suppose when I was, what 55, I was thirteen stone and hadn’t done any exercise much since I’d left school, so I was introduced to a friend to walking and stuff and running and a bit of jogging, so you know, I’m two stone lighter now than I was then.

I10 – Male, aged 68, high engager

### Perspectives on the Web-Based Intervention

Almost all participants, regardless of their level of engagement with the website, described aspects of the Web-based intervention that acted as either barriers or facilitators to use (Table 3 and Table 4).

The most commonly cited barriers related to issues with access to the intervention itself, either due to difficulties remembering the link to the site or passwords, or a perceived lack of flexibility within the website. Several participants also felt that the lifestyle advice provided was too limited and did not include sufficient options for those already achieving the goals, or with particular likes/dislikes or medical problems. Conversely, most participants commented favorably about the content of the lifestyle information provided. For many participants, the nonpreaching and nonjudgmental presentation of the lifestyle information was an important facilitator, along with the use of simple language and inclusion of up-to-date lifestyle information from a respected source. Several individuals also described how the personalized nature of the risk and lifestyle information made them feel more engaged.

A number of participants also suggested possible additions to the intervention to improve it; these included incorporating a progress chart or tracker that would allow participants to log in and update the website with their progress whilst also providing a reason to return to the website regularly to remind them of the information, and linking it with calendar applications to allow participants to add reminders to their calendars to prompt them between the scheduled sessions.

**Table 3:** Barriers to engagement.

Barrier	Representative quotations
Difficulty remembering passwords	*The thing I found most difficult was each time having to go back and try and find the password and the name thing, which I'd lost a million times down the thing, and every time I wanted to go into it so it stopped me going there so regularly because it was quite hard to go back and look at it, that might just be me!* (I37 – Female, aged 46, high engager)
Difficulty getting back into the website after clicking on additional information	*The only thing that did annoy me was when you go out… when it says, “If you want to know more about five a day” or whatever, “click here.” So, you click there and you go into the other website which sort of tells you all the information you want to know, but I couldn’t get back to the original study. So, I had to go right out and then log back in, but then it brought me back to the page I was on, so that was okay.* (I14 – Female, aged 68, high engager)
Difficulty getting back into the website for the later modules	*Well when I got the email I think last week, to do session two of the informed study I was a bit surprised there wasn’t a link on it to take me straight into it, so I had to refer back to the original email with my password and login details, I’ve now set up my own link so that’s fine, I was just a bit surprised I just didn’t press a button and it was there...but that’s a minor point and it’s something I could cope with but if I’d deleted all the information from session one I’m not sure how I would have got into it.* (I1 – Male, aged 64, high engager)
Difficulty remembering all the information	*I think the only difficulty I had was because there are quite a few pages on some of them when you go through the study, if you didn’t print them off it was difficult to remember with some of the things you might have read before, so there wasn’t like, I didn’t think there was enough of a summary at the back so when you got to the end you could then pick up all the salient points in one go and just print that off.* (I28 – Male, aged 58, high engager)
Lack of flexibility/too prescriptive	*I mean the whole thing seemed very very linear so that you started at the beginning and there really wasn’t any, you know, straight, you know, and I just felt as I say, railroaded.* (I29 – Male, aged 64, high engager)
Limited options for those with particular likes/dislikes, medical problems or already achieving the goals	*The problem is that I felt that I was only going to be able to sign up to more exercise if I honestly felt that I was going to stick to it and I hate exercise and most of the recommendations in the first module are the kind of exercise that frankly I’m not interested in doing.* (I29 – Male, aged 64, high engager) *I found the exercise one quite tricky for that because the exercise site is very much set up for people who aren’t exercising enough and it’s trying to set goals to exercise more, and it almost didn’t have any options to do the same.* (I40 – Female, aged 45, high engager)

**Table 4:** Facilitators to engagement.

Facilitator	Representative quotations
Nonpreaching nature of lifestyle information	*I found it was at a good level to read, you know, it wasn’t preaching. Sometimes you can find it’s very preachy information that comes across and, and therefore that makes me react, but when it’s just informative, saying, these are the facts, you have to now make a decision, that’s much better from my point of view.* (I20 – Male, aged 49, high engager)
Nonjudgmental	*I thought it was pretty good, it was better than I expected. I did wonder if it was going to lecture me or try to frighten me, but I thought it was quite easy to use, it was clear, the information was there and it didn’t sort of judge you or anything. So, I thought it was quite good and I did the whole thing and that was fine, yeah...If it had have been sort of condescending or over instructive, I’d have probably switched it off.* (I3 – Male, aged 57, high engager)
Links to further information	*Quite useful. Particularly, actually I’ll come back to it again, what constitutes five-a-day because you could be thinking about a large fruit or a small fruit and cooked vegetables versus non-cooked vegetables, so it was quite informative, not just the information on the site but the links it had to other information.* (I21 – Male, aged 55, high engager)
Simple language	*I found it very easy to use, very easy to digest, there was no jargon or technical terms, there was, it was just plain and simple, stating what in some cases was the obvious but put in such a way that you actually digested it.* (I36 – Female, aged 56, high engager)
Easy to navigate	*In terms of usability, it, it was quite easy to use. It was attractively laid out, it was easy to follow, yeah, I think the website was, was quite straightforward.* (I40 – Female, aged 45, high engager)
Up to date information from respected source	*I felt the quality of the advice was, it was high information content but with, with useful interpretation, and seemingly very up to date.* (I18 – Male, aged 61, high engager) *I think the INFORM stuff has helped me to, well it has a bit more authority, you know, than the [national newspapers], choose which end you want to be of the spectrum.* (I2 – Male, aged 70, high engager)
Personalized	*I know it goes to a lot of people but I feel it’s on a one-to-one, “We’ve told you this and you’ve set yourself a goal” and then however many weeks later they send you an email and it’s not hard and fast, it’s gentle but you know, “Do you remember you did this? And do you remember we said this?” And you think “Oh yeah, okay”.* (I36 – Female, aged 56, high engager)
Reminder emails	*The emails have come at the right time, they’re not coming all the time so you don’t think, “Oh I’m just going to delete it, I’m not going to read it,” it comes and you think, “Oh yeah, I haven’t thought about this,” so you read it all and you take it all in and it just revives your initial thoughts.* (I36 – Female, aged 56, high engager)

## Discussion

### Principal Results

Using a mixed-methods approach, this study demonstrates that lower engagement with a Web-based lifestyle intervention following provision of an estimate of 10-year CHD risk was associated with reporting a negative emotional reaction to the risk score, perceiving that the intervention did not provide any helpful lifestyle information, being less likely to have reported feeling an obligation to complete the intervention as part of the study and less success with prior experiences of behavior change attempts. No associations were seen between engagement with the website and the level of CHD risk or reported barriers or facilitators to health behavior change. The most commonly cited barriers to engagement were difficulty accessing the website, a perceived lack of flexibility within the website, and lack of time. Facilitators included the nonjudgmental presentation of lifestyle information, the use of simple language, and the personalized nature of the intervention.

### Strengths and Limitations

A key strength of this study is the use of a mixed-methods approach to explore associations between participants’ views expressed during the qualitative interviews and their engagement with a Web-based intervention. Unlike previous studies which have focused on differences in the clinical and sociodemographic characteristics of individuals [10-12], this approach provided us with a richer understanding of the data [27] and allowed us to compare within groups and across groups of low and high engagers to identify patterns in the qualitative and quantitative data associated with engagement with the website intervention [28]. The themes identified in the qualitative data were also the result of an inductive process using responses to questions related to the understanding of CHD risk, reactions to receiving a risk score, intentions to change behavior, and attempts at actually changing behavior rather than direct questions about engagement. Using this approach, we were able to identify associations between participants’ views and engagement that have not been reported previously.

However, the findings must be interpreted with consideration of the limitations of the study. The main limitation is that the participants were a small purposive sample selected from blood donors already taking part in another trial, so they may have had better knowledge or a more positive attitude towards healthy lifestyles than the general population. Participants were also highly educated and earning more money than the national average; their views may, therefore, not be representative of the general population and our findings may not reflect the reasons for participation among less educated or lower socioeconomic groups. By using an inductive approach guided by the data, the analysis is also limited to the topics raised during the interviews. While we identified no new themes when coding the later interviews and believe we reached data saturation, it is possible that new themes would have been present in a larger, more diverse sample. A second limitation is our measure of website engagement. We measured engagement by tracking which pages participants had accessed during the trial and considered participants high engagers if they completed all three sessions, and low engagers if they did not. While this method is better than self-report [29] as it removes the risk of recall or social desirability bias, it has been suggested that such a summative approach misses additional levels of data [30]. Tracking the time spent on each page and individual participants’ routes through the intervention would have provided more data.

### Comparison With Prior Work

Although not reported previously, the findings that engagement was lower in those who expressed negative emotions such as fear, anxiety, or worry when being asked during the interview to recall their feelings at the time they received the risk information, and in those who reported less success with prior experiences of behavior change attempts, are consistent with behavioral theory. Two widely used theories of behavior change (Protection Motivation Theory [13] and the Extended Parallel Processing Model [31]) suggest that perceptions of a health risk can cause either adaptive self-protective actions, or maladaptive self-defeating actions depending on perceptions of threat (perceived vulnerability or susceptibility, and perceived severity) and efficacy (response efficacy or the perceived effectiveness of the recommended actions; and self-efficacy, a person's belief about his or her ability and capacity to achieve the recommended changes). These models suggest that if the health threat is believed to be inevitable or unrealistic, or people do not believe in their own ability to change their behavior, instead of engaging in health protective behaviors, people may experience thoughts of fatalism or hopelessness and engage in psychological defense mechanisms such as avoidance and denial. Together with the findings in this study, this suggests that providing individuals with risk information may potentially decrease engagement in prevention activities through maladaptive behaviors, and that different approaches may be needed depending on an individual’s prior beliefs and understanding of their own risk. In this study, participants viewed their risk online, but when risk is provided in face-to-face consultations it is easier to address negative emotional reactions at the time, which may reduce maladaptive coping strategies and improve subsequent engagement.

The findings that perception that there was little or no helpful lifestyle information provided by the intervention was associated with not completing the intervention, and a third of those who were high engagers with the Web-based intervention reported feeling an obligation to complete the intervention as part of the study, are also consistent with reports on participation in research. Two of the key motivators for taking part in clinical trials are a willingness to help others and contribute towards furthering medical knowledge, and perceiving some benefit (and/or no significant disadvantage) for themselves [32-34]. A number of participants completed the intervention out of a sense of duty, with their focus less on improving their own health and more about contributing to the improvement of others’ health. This is not entirely surprising, but does have implications for the generalizability of the findings of Web-based interventions outside of trial settings.

In addition to these individual-level factors associated with engagement with the intervention, this study also highlights a number of features of Web-based interventions that can act as either barriers or facilitators. The most common barriers that were reported related to difficulties with access to the intervention itself, such as forgetting the link to the website or passwords [35]. Simple amendments, such as including links to the website in every email correspondence and incorporating an automatic password reset option for forgotten passwords, may therefore increase engagement. Similarly, being mindful when developing interventions to include a wide range of options for those with particular needs (where possible) may help to retain the interest of some individuals. While one style is unlikely to suit everyone, the findings of this study also suggest that in the context of behavior change interventions, presenting lifestyle information in a nonjudgmental and nonpreaching style is appreciated. This finding is echoed in studies reporting why people chose not to take up the offer of cardiovascular screening within the NHS Health Check program, in which a number described not wishing to be *told off* as a contributory factor [36-38].

### Conclusions

In the context of a growth of interest in scalable interventions, where small effect sizes across large numbers of individuals have the potential to impact health at the population level, this study has a number of implications for clinicians involved in communicating risk of disease and providing lifestyle advice, and those developing Web-based interventions. Our findings suggest that tailoring Web-based health behavior change interventions to take account of participants’ prior perceptions of their risk, any earlier attempts at behavior change, and their current knowledge of health behaviors may improve engagement. These approaches could be achieved by presenting risk in different visual or verbal formats, using behavior change techniques targeted at improving self-efficacy for those with previous failed attempts at behavior change, and providing information in a stepwise manner with more complex information available for those with greater baseline knowledge. Seeking to prevent or address negative emotions at the time of delivery of risk information by providing endorsement of the risk information and Web-based intervention at the time of referral or provision of risk, may also reduce subsequent maladaptive coping strategies. Developing or recommending interventions that take account of difficulties with access and perceived lack of flexibility by having simple password reminder systems and clear navigation, whilst continuing to present lifestyle information in a nonjudgmental way using simple language, may also increase engagement and reduce attrition.

## Abbreviations

CHD: coronary heart disease
CVD: cardiovascular disease
INFORM: Information and Risk Modification
NHS: National Health Service
